# Antimicrobial Activity of the Iron-Chelator, DIBI, against Multidrug-Resistant Canine Methicillin-Susceptible *Staphylococcus pseudintermedius*: A Preliminary Study of Four Clinical Strains

**DOI:** 10.3390/pathogens11060656

**Published:** 2022-06-07

**Authors:** Francesca Paola Nocera, Giuseppe Iovane, Luisa De Martino, Bruce E. Holbein

**Affiliations:** 1 Department of Veterinary Medicine and Animal Production, University of Naples Federico II, 80137 Naples, Italy; francescapaola.nocera@unina.it (F.P.N.); iovane@unina.it (G.I.); 2 Task Force on Microbiome Studies, University of Naples Federico II, 80137 Naples, Italy; 3 Fe Pharmaceuticals (Canada) Inc. (Formerly Chelation Partners Inc.), #58 The Labs at Innovacorp, Life Sciences Research Institute, 1344 Summer Street, Halifax, NS B3H 0A8, Canada; bholbein@cpintercept.com

**Keywords:** antimicrobial resistance, *Staphylococcus pseudintermedius*, iron chelator, DIBI

## Abstract

*Staphylococcus pseudintermedius* is an important opportunistic pathogen causing various infections in dogs. Furthermore, it is an emerging zoonotic agent and both multidrug-resistant methicillin-resistant *S. pseudintermedius* (MRSP) as well as methicillin-susceptible (MSSP) strains represent an important therapeutic challenge to veterinary medicine and pose a potential threat to human health. We tested representative *S. pseudintermedius* clinical strains from dogs suffering from otitis externa for their susceptibilities to a panel of 17 antimicrobials compared to DIBI. DIBI, unlike antibiotics, is a novel water-soluble hydroxypyridinone-containing iron-chelating agent that deprives microbes of growth-essential iron and has been previously shown to inhibit methicillin-resistant *Staphylococcus aureus* (MRSA). We also characterised the strains according to whether they harbour key antibiotic resistance genes. The strains each displayed multiple antimicrobial resistance patterns; all were negative for the *mec*A gene and possessed the *tet*K and *tet*M genes, but they varied as to their possession of the *erm*B gene. However, all the isolates had similar susceptibility to DIBI with low MICs (2 µg/mL or 0.2 µM). Because the four MSSPs were equally susceptible to DIBI, subject to confirmation with additional strains, this could provide a potential non-antibiotic, anti-infective alternative approach for the treatment of antimicrobial-resistant canine *S. pseudintermedius* otitis.

## 1. Introduction

*Staphylococcus pseudintermedius (S. pseudintermedius)*, an emerging zoonotic agent of canine origin, is an important opportunistic pathogen causing various infections in dogs, such as otitis externa, pyoderma, and wound infections [1,2,3]. Multidrug-resistant (MDR) methicillin-resistant *S. pseudintermedius* (MRSP) and methicillin-susceptible *S. pseudintermedius* (MSSP) strains represent an important problem in animal health and an increasing therapeutic challenge in veterinary medicine [4]. Importantly, *S. pseudintermedius* has also been isolated from human infections; it shares a number of similarities with *S. aureus* and there is growing evidence for both the horizontal and vertical genetic transfer of antibiotic resistance in staphylococci [5]. This further increases concerns over antimicrobial-resistant (AR) *S. pseudintermedius* in companion animals, i.e., given the potential spread of antibiotic-resistant infections to humans.

The antimicrobial resistance of bacterial pathogens is one of the most urgent threats to public health and the widespread emergence of MDR bacterial pathogens is a problem of global dimensions. The regulatory pressure to reduce antimicrobial use; the ever-increasing incidence of MDR bacterial strains among companion animals suffering from otitis externa, pyoderma, and other infections; and the progressively limiting therapeutic options both in veterinary and human medicine underline the need for new alternative treatment approaches in order to prevent and control staphylococcal infections [6,7,8].

Among new therapeutic strategies, one is based on the use of a combination of antibiotic therapies, which can broaden the antibacterial spectrum, address polymicrobial infections, and has a potential synergistic action [9]. Another strategy is the use of adjuvants able to act in concert with the known conventional antimicrobial agents, thus enhancing their activity, especially against resistant isolates [10]. A non-antibiotic treatment approach is particularly interesting as are agents that can improve responses to conventionally used antibiotics. Alternative treatments for bacterial infections, such as phage therapy, probiotics and prebiotics, antibacterial peptides, nanoparticles, etc., represent noteworthy ongoing developments. Indeed, alternative non-antibiotic treatment strategies need to be explored to ensure that a robust pipeline of effective therapies is available to both human and veterinary medicine [11].

**Figure 1:**
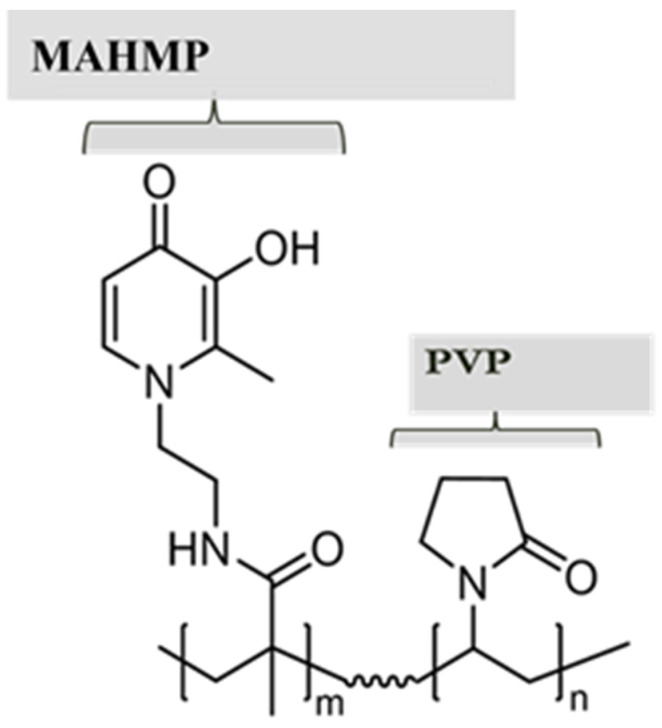
DIBI (first-in-class non-antibiotic Fe^+3^ -sequestering anti-infective co-polymer) chemical structure.

Microbial pathogens have irreplaceable requirements for iron for their growth and virulence and iron withdrawal agents can provide a broad-based approach to restrict pathogen growth and also work in conjunction with conventional antibiotics to enhance efficacy for AR isolates [12]. DIBI, a novel water-soluble hydroxypyridinone-containing iron-chelating 9kDa polymer, provides a potential new antibacterial treatment by denying pathogens iron as needed for their growth (Figure 1).

Previous studies have shown that DIBI is effective against various Gram-positive and Gram-negative bacteria (examples, *Staphylococcus aureus* and *Acinetobacter baumannii*) and fungi (example, *Candida albicans*) [13,14,15,16]. DIBI has been purposely developed to bolster the existing innate iron withdrawal host defences and has been shown to be orally and systemically non-toxic when administrated at repeated high dosages as well as to the ear canals of healthy dogs [12].

In this study, the antibacterial activity of DIBI against four multidrug-resistant MSSP clinical strains was evaluated.

## 2. Results

### 2.1. S. pseudintermedius Identification

**Figure 2:**
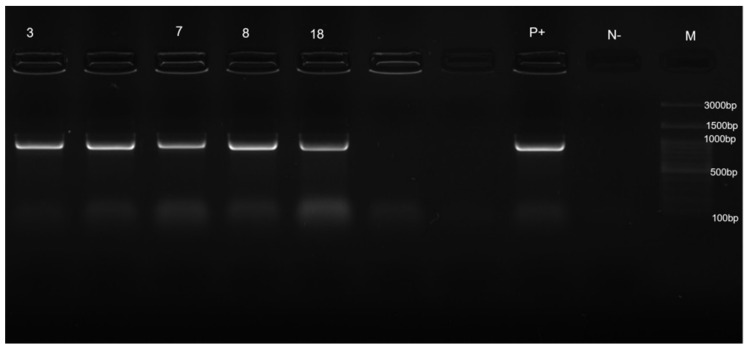
PCR for detection of species-specific *nuc* gene. Data from one of three experiments are shown. Lanes 3, 7, 8, 18, strains tested; lane P+, positive control; lane N−, negative control; lane M, 100-bp DNA ladder.

**Figure 3:**
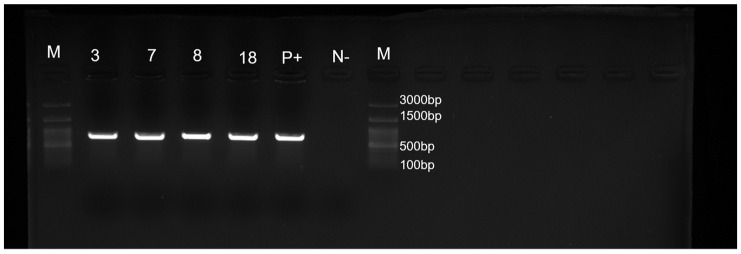
PCR for detection of species-specific *hlb* gene. Data from one of three experiments are shown. Lanes 3, 7, 8, 18, strains tested; lane P+, positive control; lane N−, negative control; lane M, 100-bp DNA ladder.

*S. pseudintermedius* isolated strains were identified by MALDI TOF MS with a log (score) ≥ 2.0. In all four selected *S. pseudintermedius* strains, the proteomic identification using MALDI TOF MS was then confirmed by molecular profiling using PCR, to detect whether they were harbouring the species-specific *nuc* (Figure 2) and *hlb* (Figure 3) genes.

### 2.2. Phenotypic and Genotypic Characterization of S. pseudintermedius Antibiotic Resistance Profiles

**Figure 4:**
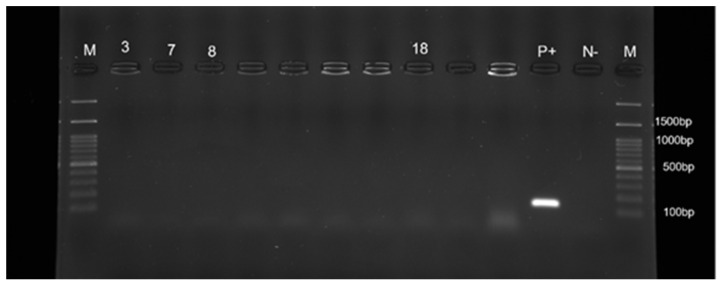
PCR for detection of *mec*A gene. Data from one of three experiments are shown. Lanes 3, 7, 8, 18, strains tested; lane P+, positive control; lane N−, negative control; lane M, 100-bp DNA ladder.

The four *S. pseudintermedius* strains were classified as MSSP, being negative for the *mec*A gene (Figure 4) and phenotypically susceptible to oxacillin. However, the antimicrobial susceptibility results obtained by Kirby–Bauer disk diffusion testing on Mueller–Hinton agar (Liofilchem, Teramo, Italy), revealed a complete resistance to amoxicillin-clavulanate and ampicillin (100%) for all MSSP strains. The highest resistance rates to selected non-*β*-lactam antimicrobials were tetracycline (100%), followed by erythromycin, sulfamethoxazole/trimethoprim, and streptomycin (75% for all three). Importantly, the MSSP strains were found to be multidrug-resistant with resistance to at least three different antimicrobial classes (Table 1). Interestingly, none of the MSSP strains were resistant to ceftriaxone, ciprofloxacin, enrofloxacin, linezolid, or vancomycin.

The phenotypically tetracycline-resistant MSSP strains harboured *tet*K and *tet*M genes in the association. The erythromycin-resistant gene *erm*B was found in the MSSP isolates, which were phenotypically erythromycin-resistant. The distribution of the *tet*K and *tet*M genes, and the *erm*B genes among the MSSPs is shown in Table 1.

### 2.3. Susceptibility of S. pseudintermedius Strains to DIBI

All the *S. pseudintermedius* strains displayed high susceptibilities to DIBI with a MIC of 2 µg/mL at 24 h (Table 1). Furthermore, DIBI susceptibility was not linked to antimicrobial susceptibility or resistance for these strains.

## 3. Discussion

We have shown that four canine strains of *S. pseudintermedius* obtained from the University Veterinary Teaching Hospital in Naples, Italy, had multidrug resistance (MDR) profiles, being resistant to antimicrobial agents that are frequently prescribed for dogs with both MRSP and MSSP infections. Our findings are consistent with those of others, showing the broad, worldwide, increasing prevalence of antimicrobial-resistant *S. pseudintermedius* strains in dogs [17,18,19]. In addition, MSSP strains have shown increased resistance to most of the antimicrobials currently licensed for use in pets [3,4,20,21,22,23,24]. Our MSSP strains were all resistant to antimicrobial agents commonly used to treat canine infections, such as penicillinase-labile penicillins, tetracycline, aminoglycosides, macrolides, and sulfamethoxazole/trimethoprim. Worryingly, these MSSP strains were found to be multidrug-resistant, with resistance to at least three different antimicrobial classes. However, it is worth noting that none of these MSSP strains showed resistance to vancomycin and linezolid. The use of these agents in veterinary medicine is controversial and no longer permitted for use in dogs in the European Union since they represent the last resort reserve antibiotics for treating severe infections caused by methicillin-resistant staphylococci in human medicine. In this regard, the high susceptibility of our strains to DIBI is interesting and this warrants additional studies using a larger number of strains.

An interesting recent finding may in part explain the increasing MDR *S. pseudintermedius* prevalence. MDR *S. pseudintermedius* strains have been shown to be strong biofilm growers in comparison to their non-MDR counterparts [25]. A capacity for biofilm growth is a potential colonisation attribute and it seems consistent with carriage and growth on the skin and other epithelial tissues of dogs, i.e., biofilm growth might be expected to favour colonisation and carriage at these sites. Given the above and the linkage of biofilm growth to MDR in *S. pseudintermedius* [25], it seems plausible that the niche habitats of dogs that favour biofilm growth of *S. pseudintermedius* may be positively selecting not only for biofilm competent but also for MDR isolates. The possibility that MDR of *S. pseudintermedius* favours its colonisation of dogs by conferring a biofilm growth advantage warrants further investigation given its important implications for increased canine and possibly human MDR MSSP and MRSP infections.

We have shown that the isolated MSSP strains had similar susceptibilities to the iron-withdrawal chelator DIBI. Iron withdrawal has also been shown to disrupt the staphylococcal biofilm growth of coagulase-negative isolates, increasing their susceptibility to antibiotics [26]. DIBI should be further investigated for its abilities to impede biofilm growth and enhance antibiotic efficacy for both MRSP and MSSP strains.

The issue of the increased zoonotic transmission to and from companion animals and humans is especially concerning given that the co-carriage of both MRSP and methicillin-resistant *S. aureus* (MRSA) can occur and there is a potential exchange of AR genes among these different staphylococci [5]. Antimicrobial-resistant infection is one of the most urgent threats to public health and the increasingly limited therapeutic options in both veterinary and human medicine underline the need for alternative anti-infectives. In this study, we have investigated the novel iron-withdrawal agent DIBI for its potential to inhibit the growth of MSSP. DIBI was strongly inhibitory to multidrug-resistant MSSP strains, exhibiting very low MIC values of 2 µg/mL (0.2 µM) for each of the strains. In a separate USA-based clinical study, DIBI performed comparably to a standard veterinary otitis medicine containing two antibiotics (miconazole and polymyxin B) for the treatment of dog patients presenting at veterinary clinics with otitis externa [27]. In that study, staphylococci represented 30% of the isolates cultured from the 28 target ears and of these, *S. pseudintermedius* was the most prevalent. Taken together, our results indicate that DIBI could be a potential non-antibiotic, anti-infective, alternative therapeutic approach for treating canine *S. pseudintermedius* infections caused by MSSP.

Parquet et al. [14] demonstrated that DIBI was also strongly inhibitory for a diverse group of *S. aureus* isolates with low MICs regardless of the isolate’s animal origin (human, cattle, or dogs) and irrespective of their antibiotic-resistant (MRSA) characteristics. In that study, DIBI also demonstrated anti-infective activity for experimental MRSA surficial skin infections in mice. Given other studies have shown DIBI to have activity for both AR Gram-negative bacteria [15] and AR yeast [16], DIBI appears to have broad anti-microbial and anti-infective activities, which are independent of the antimicrobial resistance profile of the particular bacterial pathogen. Consistent with this, the isolates we characterised all possessed the *tet*K and *tet*M genes but varied as to the presence of the *erm*B gene, yet all were equally susceptible to DIBI. A limitation of our study is the low number of strains tested, but further studies are planned against a larger number of both MDR MSSP and MRSP strains. Despite its relatively small sample size, this study demonstrated the importance of conducting research related to possible alternative antimicrobial approaches in order to counteract the impact of MDR *S. pseudintermedius* infections on animal health. Thus, subject to confirmation with additional strains, DIBI could be considered a non-antibiotic alternative therapy in veterinary medicine against MDR canine otitis externa MSSP strains.

## 4. Materials and Methods

### 4.1. Identification of S. pseudintermedius Strains

Four clinical canine otitis *S. pseudintermedius* strains identified as #s 3, 7, 8, and 18, were selected from the bacterial culture collection of the Microbiology Laboratory of the Department of Veterinary Medicine and Animal Production, University of Naples Federico II, Italy. The strains had been obtained from auricular swabs of four dogs referred for otitis externa to the University Veterinary Teaching Hospital called Ospedale Veterinario Universitario Didattico (OVUD) in the above-mentioned department. Swabs were inoculated onto Columbia CNA agar with 5% sheep blood and on mannitol salt agar (MSA) (Liofilchem, Teramo, Italy) and incubated aerobically at 37 °C for 24–48 h. *S. pseudintermedius* ATCC^®^ 49444^TM^ was used as a positive control for identification. Suspected *S. pseudintermedius* strains were first identified by using standard, rapid screening techniques: colony morphology, β-haemolysis on Columbia CNA agar, absence of mannitol fermentation on MSA, cellular morphology (after Gram’s staining method), catalase test. Additionally, each mannitol salt negative colony was also subjected to staphylocoagulase (tube coagulase) reaction (Oxoid, Ltd., Hampshire, UK) to confirm their capacity to produce coagulase enzyme. These isolates were then further identified by matrix-assisted laser desorption ionisation time-of-flight mass spectrometry (MALDI-TOF MS) analysis (Bruker Daltonics, Bremen, Germany). For MALDI-TOF MS, fresh colonies grown on Columbia CNA agar were used.

Molecular profiling using a species-specific *nuc* gene [28] and a species-specific *hlb* gene [29] was performed by single PCR to confirm the identification of *S. pseudintermedius*. For the genotypic characterization of the strains, *S. pseudintermedius* ATCC^®^ 49444^TM^ was again used as a positive control. DNA extraction from the isolates was performed using the commercial Isolate II Genomic DNA Kit (Bioline, London, UK) following the manufacturer’s instructions. Bacterial DNA was stored at −20 °C and used for further studies. Primer sequences, amplicon sizes, and amplification programs are reported in Table 2.

### 4.2. Antibiotic Susceptibility Testing of S. pseudintermedius Strains

Based on the Kirby–Bauer disc diffusion susceptibility method, all the identified *S. pseudintermedius* strains were tested with the following 17 antimicrobials: amoxicillin-clavulanate (AMC, 20/10 μg), ampicillin (AMP, 10 μg), ceftriaxone (CRO, 30 μg), clindamycin (CD, 2 μg), ciprofloxacin (CIP, 30 μg), erythromycin (E, 15 μg), enrofloxacin (ENR, 5 μg), gentamicin (CN, 10 μg), imipenem (IMI, 10 μg), linezolid (LNZ, 30 μg), oxacillin (OX, 1 μg), penicillin (P, 10 IU), streptomycin (S, 10 μg), sulfamethoxazole-trimethoprim (SXT, 1.25/23.75 μg), tobramycin (TOB, 10 μg), tetracycline (TE, 30 μg), and vancomycin (VA, 30 μg). The isolates were classified as susceptible (S) or resistant (R) according to the Clinical and Laboratory Standards Institute [30] and European Committee on Antimicrobial Susceptibility Testing [31] guidelines. For streptomycin and vancomycin breakpoints, those recommended by the French Society for Microbiology (http://www.sfm-microbiologie.fr [accessed on 10 March 2017]) were employed. Multidrug resistance was defined according to Magiorakos et al. [32] for *S. pseudintermedius* strains showing resistance to at least three different antibiotic classes.

### 4.3. Genotypic Characterization of Antibiotic Resistance

After bacterial DNA extraction from the *S. pseudintermedius* strains as described above, genetic profiles of antibiotic resistance using single PCR for *mec*A [33] gene and multiplex PCR for *tet*K and *tet*M [34] and *erm*A, *erm*B, and *erm*C [35,36] genes were systematically performed. Moreover, single PCR was performed for each tetracycline- and erythromycin-resistant gene.

*S. aureus* ATCC^®^ BAA44^TM^ was used as *mec*A positive control and PCR for tetracycline- and erythromycin-resistant genes were validated using in-house positive- and negative-control *S. aureus* strains, for which both phenotypic and genomic antibiotic resistance profiles were available. Primer sequences, amplicon sizes, and amplification programs are reported in Table 3.

### 4.4. In Vitro Susceptibility of Clinical Canine S. pseudintermedius Strains to DIBI

DIBI, a water-soluble, linear co-polymer functionalised with 3-hydroxypyridin-4-one (HPO) chelators that selectively and strongly bind iron (III) in biological environments [12], was tested against four clinical canine otitis *S. pseudintermedius* strains. *S. pseudintermedius* strains were routinely cultured from glycerol frozen stocks (−80 °C) and maintained on trypticase soy agar (TSA). To test DIBI activity, liquid cultures were grown in Roswell Park Memorial Institute Medium 1640 (RPMI, Sigma-Aldrich) supplemented with 2% (*w*/*v*) glucose, buffered with 0.165 M 3-(N-morpholino)-propanesulfonic acid (MOPS) for 18–24 h at 37 °C with shaking. RPMI medium was chosen for this study because it contains a relatively low yet sufficient iron content (approx 0.1 µM Fe) and this, therefore, better emulates in vivo iron-availability conditions [14]. RPMI-grown cultures were used to prepare inocula for MIC determinations.

### 4.5. DIBI Minimum Inhibitory Concentration (MIC) Determinations

DIBI stocks were prepared in RPMI (200 mg/mL). All stock solutions were filter-sterilised (0.2 µm filter) before use. Unlike antibiotics, DIBI interrupts microbial iron supply and, therefore, affects many iron-dependent targets at the level of DNA, protein, and lipid synthesis as well as energy production and defence.

MIC determinations were determined using the broth microdilution method [31] in 96-well round-bottomed plates (Life Sciences, Halifax, NS, Canada). RPMI was the assay medium and diluent testing *S. pseudintermedius* strains, as already described for other bacteria of animal origin [14,37].

The MIC for DIBI was determined and for this DIBI was diluted in RPMI with serial 1/2 dilutions made covering the ranges 0.06–128 µg/mL. To prepare the serially diluted 96-well plates, RPMI was added first to each well. Next, a similar volume of DIBI at a concentration equal to twice the highest concentration (2×) desired was added to the well. Then, the contents of the wells were mixed and serially diluted across the row to achieve the dilution series.

For inoculum preparation, RPMI overnight *S. pseudintermedius* cultures were diluted in their respective fresh media to an optical density (OD 600 nm) of 0.1 and MIC plates were inoculated to a final dilution of 1/200. Negative and positive controls were included in parallel. All MIC plates were incubated at 35 °C for 24–72 h and then read as to MIC. MIC value was defined as the lowest concentration of chelator required to inhibit visible growth at 24 h incubation.

## 5. Conclusions and Future Investigations

The results reported above suggest that DIBI may be considered a potential therapeutic agent to treat otitis infection caused by MDR MSSP strains. Specifically, the low DIBI MICs (2 µg/mL or 0.2 µM) recorded for the multidrug-resistant MSSP strains support the need for further investigation of DIBI’s antimicrobial activity against antimicrobial-resistant canine otitis MRSP to confirm its potential as an alternative therapeutic approach for treating *S. pseudintermedius* otitis. Furthermore, the known ability of *S. pseudintermedius* to form biofilms and the biofilm link to resistance, warrant further studies on the DIBI inhibition of *S. pseudintermedius* biofilm growth.

Awareness, current research, and the comprehensive management of infections are required by veterinarians not only to support infected companion animals but also to limit the spread and prevent the establishment of this highly drug-resistant and zoonotic pathogen in veterinary facilities and the community.

## Figures and Tables

**Table 1:** Antimicrobial resistance profiles and DIBI MIC for *S. pseudintermedius* strains.

Strain	Key Antibiotic Resistance	*mec*A Gene Detection	*erm* and *tet* Genes	DIBI MIC µg/mL
3	AMC, AMP, CD E, P, S, SXT, TE	-	*erm*B, *tet*K, *tet*M	2
7	AMC, AMP, E, CN, P, S, SXT, TE, TOB	-	*erm*B, *tet*K, *tet*M	2
8	AMC, AMP, P, SXT, TE	-	*tet*K, *tet*M	2
18	AMC, AMP, E, IMI, P, S, TE	-	*erm*B, *tet*K, *tet*M	2

Antimicrobial agents: AMC: amoxicillin-clavulanate, AMP: ampicillin, CD: clindamycin, E: erythromycin, CN: gentamicin, IMI: imipenem, P: penicillin, S: streptomycin, SXT: sulfamethoxazole-trimethoprim, TOB: tobramycin, TE: tetracycline.

**Table 2:** Primer sequences, amplicon sizes, amplification programs of *nuc* and *hlb* genes.

Gene	Primer Sequences (5′–3′ Sense and Antisense)	Amplicon Size (bp)	Amplification Program	Reference
*nuc*	F: TRGGCAGTAGGATTCGTTAA R: CTTTTGTGCTYCMTTTTGG	926	94 °C 5 min; 94 °C 30 s, 58 °C 60 s, 72 °C 90 s, for 30 cycles; 72 °C 5 min.	[28]
*hlb*	F: GACGAAAATCAAGCGGAA R: TCTAAATACTCTGGCGCAC	734	94 °C 2:30 min; 94 °C 30 s, 56 °C 30 s, 72 °C 1 min, for 30 cycles; 72 °C 10 min.	[29]

**Table 3:** Primer sequences, amplicon sizes, amplification programs of *mec*A, *tet*, and *erm* genes.

Gene	Primer Sequences (5′–3′ Sense and Antisense)	Amplicon Size (bp)	Amplification Programs	References
*mec*A	F: TCCACCCTCAAACAGGTGAA R: TGGAACTTGTTGAGCAGAGGT	139 bp	94 °C 5 min; 94 °C 30 s, 55 °C 40 s, 72 °C 30 s, for 30 cycles; 72 °C 5 min	[33]
*tet*K	F: GTAGCGACAATAGGTAATAGT R: GTAGTGACAATAAACCTCCTA	360 bp	94 °C 15 s; 94 °C 1 min, 52 °C 1 min, 72 °C 90 s, for 30 cycles; 72 °C 5 min	
*tet*M	F: AGTTTTAGCTCATGTTGATG R: TCCGACTATTTAGACGACGG	1862 bp		[34]
*erm*A	F: TCTAAAAGCATGTAAAAGAA R: CTTCGATAGTTTATTAATATTAGT	645 bp	94 °C 2 min; 94 °C 1 min, 55 °C 1 min, 72 °C 90 s, for 30 cycles; 72 °C 5 min	
*erm*B	F: GAAAAGGTACTCAACCAAATA R: AGTAACGGTACTTAAATTGTTTAC	639 bp		[35,36]
*erm*C	F: TCAAAACATAATATAGATAAA R: GCTAATATTGTTTAAATCGTCAAT	642 bp		

## Data Availability

Not applicable.
